# Adoption of antenatal care conversation mapping among health care providers in Saudi Arabia: Application of the diffusion innovation theory

**DOI:** 10.1371/journal.pone.0286656

**Published:** 2023-06-08

**Authors:** Anwar Alhashem, Bayader A. Alotaiby, Rahaf B. Al thobaiti, Mudhi M. Almaktoomi, Shahad I. Alzahrani, Alia A. Albaiz, Basil H. Aboul-Enein, Nada Benajiba

**Affiliations:** 1 Department of Health Sciences, College of Health and Rehabilitation, Princess Nourah bint Abdulrahman University, Riyadh, Saudi Arabia; 2 Department of Epidemiology and Biostatistics, Health Science Research Centre, Princess Nourah bint Abdulrahman University, Riyadh, Saudi Arabia; 3 Faculty of Public Health and Policy, London School of Hygiene & Tropical Medicine, London, United Kingdom; 4 Department of Basic Health Sciences, Deanship of Preparatory Year, Princess Nourah bint Abdulrahman University, Riyadh, Saudi Arabia; Nazarbayev University School of Medicine, KAZAKHSTAN

## Abstract

**Aim:**

To measure the factors influencing on the adoption of antenatal care conversation mapping among health care providers in Riyadh (Saudi Arabia), using the diffusion innovation theory.

**Methods:**

88 healthcare providers (Riyadh) were recruited using a non-probability convenient sampling technique were trained on how to use a newly developed antenatal care conversation map. Data was collected by self-administrated questionnaire on health education services, adoption of conversation map and diffusion of innovation variables. The JMP statistical software from SAS version 14 was used to perform data analysis.

**Results:**

Printable tools were most common as used by 72.7% of participants and 83.0% of them did not hear about conversation map. The total mean score of diffusion of innovation variables showed was in general high. The total mean score of relative advantage and observability was high in participants aged between 40 to less than 50 years, while the total mean score of compatibility, complexity, and trialability was high in participants aged from 50 years and more. Significant differences were obtained in both compatibility and trialability considering the health educators specialty, p = 0.03 and p = 0.027 respectively. The linear correlations between diffusion of innovation variables was significantly positive (p-value <0.01).

**Conclusion:**

All of diffusion of innovation variables were positive as per the opinion of the participants. Applying the conversation map on other health topics in Saudi Arabia and other Arabic-speaking countries is warranted. Measuring and evaluating the adoption rate of conversation mapping among health care providers on other health topics should be explored.

## Introduction

Principles of women’s health education are designed to facilitate the prevention of diseases in women. This includes diagnosis, health screening and maintenance of cases that are serious, more common, and prevalent among women [1]. In developing countries, more than a half million women die and many more suffer from disabilities due to pregnancy related morbidities every year. For example, 20% of global maternal deaths was identified in India, even with public health improvements. Interventions involving antenatal care could serve to prevent maternal deaths [2,3]. It is widely recognized that high quality antenatal care is a crucial element towards maternal healthcare. According to research, poor pregnancy outcomes are highly associated with substandard prenatal, intrapartum, and postnatal care [4]. Maternal health, nutrition, availability, and usage of health care are all risk factors for perinatal death [5]. Routine antenatal care is defined as the care provided by medical and health practitioners and allied health professionals to ensure the best health conditions and outcomes for pregnant women and their developing fetuses during pregnancy. Issues with visiting prenatal healthcare services were highlighted by mothers and physicians, who found several barriers to accessing prenatal care facilities. These factors were divided into the following three categories: Lack of transportation, low parental education, and inadequate healthcare facilities [6]. One of the many essential components involved in antenatal care is patient health education and health promotion.

In both developing and developed countries, patient education is considered an essential determinant of health leading to improved utilization and understanding of health instructions [7]. According to Ghafoor’s et al. [8] study, conversation mapping was used for diabetes, and it contained multiple stations, each station explaining different aspect of the topic. The stations were presented as interrelated sequential pictures. Some of the advantages of conversation mapping include: 1.) information guidance from the health care provider and 2.) patient capability to make informed decisions that affects their self-management [9]. As an additional activity, patients were given cards labelled as ‘my goals’ card in which they wrote goals they aimed to reach or maintain, for example, weight and blood sugar level. Patients discussed with each other and with health care providers to share their experiences [8]. These goals cards motivated patients to set their goals and help them commit to these goals towards positive health outcomes [9]. To develop health education materials, educators follow an organized process starting with setting up the goals for education, followed by literature review and focus group discussion, content selection, designing the rough draft, seeking expert comments and validation. To establish a management plan directed towards patient education care, health care practitioners can use an evaluation questionnaire and efficient health education tools to help them [10].

The diffusion of innovation theory focuses on the process that occurs when people adopt a new idea, philosophy, practice, product etc [11]. The perceived attributes of the innovation have five factors or variables that influence the adoption rate. Those variables are relative advantage, compatibility, complexity, trialability and observability [12]. Relative advantage states that the only way to adopt an innovation is when the product is better than what supersedes it. The advantages of innovation can be social, utilization, economic and so on. Compatibility variables have two meaning: first, when innovation is compatible with users’ beliefs, norms, and perceived needs. Second, when innovation can be modified to fit possible adopters needs and context. Complexity variable, easy used innovations will have more chance to be adopted, while innovations with complex use will have less chance to be adopted. Also, when the innovations broken down into more than one part the chance of adoption will increase. Trialability, if the innovations were experimented before than it will be adopted faster. Observability, when the innovation benefits are noticed and apparent to others [11].

In Saudi Arabia, studies involving antenatal care health education efforts are limited. Saudi women require crucial health education involving various maternal health aspects, including psychological health during pregnancy [13]. In Saudi Arabia, some patient educational tools used for antenatal care were either traditional materials like pamphlets and videos or were not evidence-based [14]. Additionally, few patient educational tools were developed and evaluated [9,15]. It is noted that conversation mapping has been used in more than 110 countries and translated into 35 languages primarily on diabetes education [8]. The utilization of conversation mapping in an Arabic-speaking country such as Saudi Arabia is relatively new. Therefore, the diffusion of innovation theory will help to predict the use of conversation map among health care providers during patient education sessions. For this study, an antenatal care conversation map was designed as well as an assessment tool to measure the factors that influence the adoption of the conversation map. The research question set for this study was to what extent the use antenatal care conversation mapping among health care providers in Riyadh (Saudi Arabia) will be adopted based on evaluating the variables of the diffusion innovation theory.

## Methods

### Study design and subjects

This cross-sectional was conducted at five different locations in Riyadh City: 1-Saudi Ministry of Health, 2- King Abdullah bin Abdulaziz University Hospital, 3- Security Forces Hospital, 4- Primary Health Care centers at Alnarjis district and 5- Primary Health care at Alwadi districts. Selection was based on ease accessibility and reach out of health care providers. The study was carried out over 7 months from October 2019 to April 2020. The study population was health care providers who have a capacity for conducting patient education activities, including pregnant women.

Data collection was performed after obtaining the approval from Institutional Review Board at Princess Nourah bint Abdulrahman University (Number of the IRB: 200016), and from hospitals, primary health care centers and Saudi Ministry of Health. Before signing the consent to part of the study, participants were informed about the objective of the study, the nature of their participation. They were assured about data confidentiality, anonymity, voluntary participation and the right to withdraw at any time.

The study included 88 health care providers who were recruited using a non-probability convenient sampling technique. The inclusion criteria involved health care provider with the capacity to provide health education to the patient.

### Educational material

#### The antenatal conversation map

**Fig 1:**
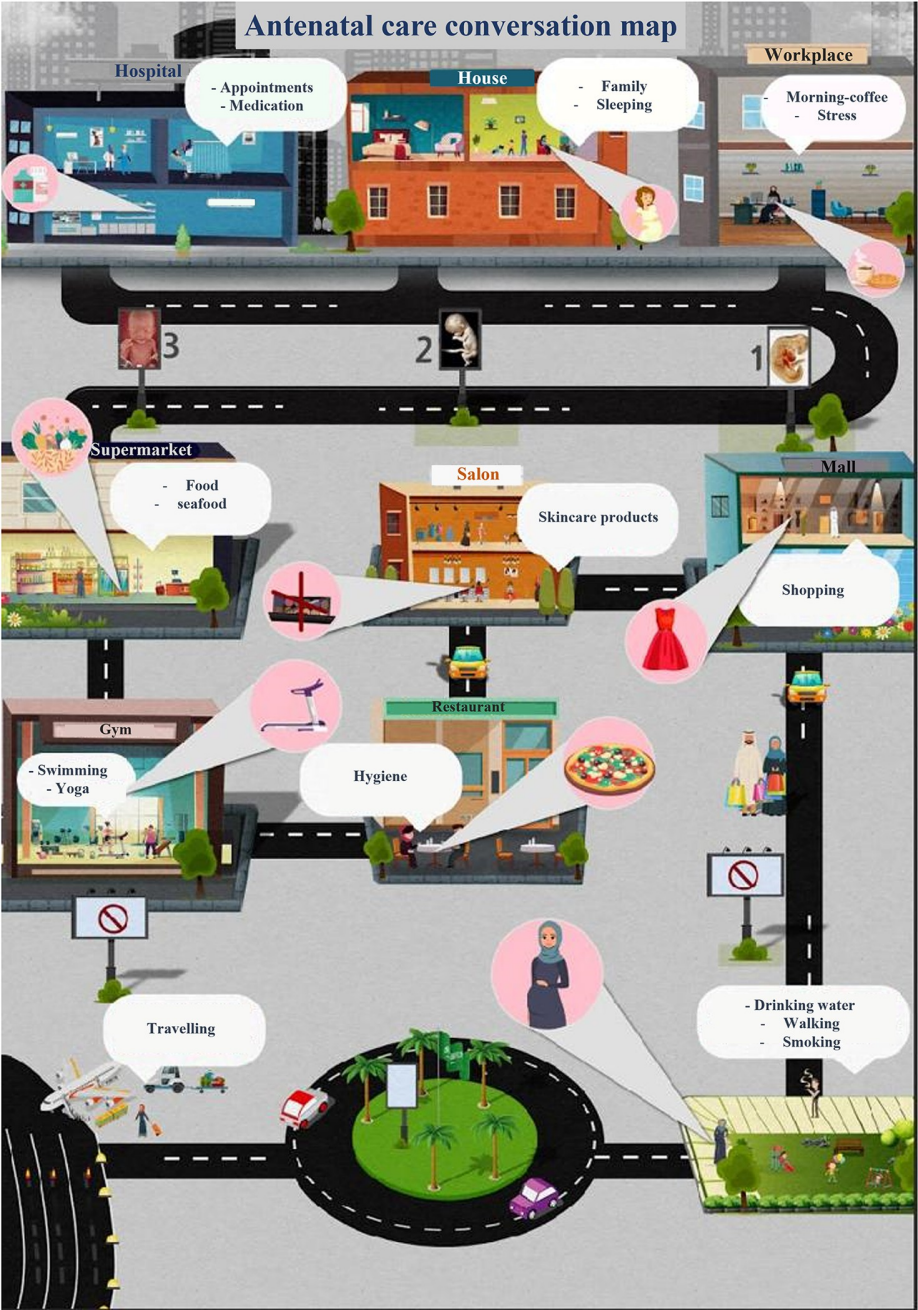
Antenatal care conversation map developed by the researchers.

The antenatal care conversation map is a new educational tool, which was designed and developed by the researchers (Fig 1). This map comprised ten stations covering everyday lifestyle activity by pregnant women. Each station represents a place with specific recommendations, as explained below:

1. **Station #1 *the hospital*:** It provides important information pregnancy trimesters, medical tips that pregnant women might need, such as the tests to be performed and the allowed medications during pregnancy. It also emphasizes on the importance of medical follow up during pregnancy.
2. **Station #2 *the house (home)***: It highlights the importance of the comfort of the pregnant woman, the embryo care and how to balance with caring about her family (*if she has*).
3. **Station #3 *the workplace***: It provides advices about work commute, argonomics, what to do during work, how to handle the morning nausea, importance of taking breakfast regularly.
4. **Station #4 *the supermarket***: It describes the most important nutrients for the pregnant, how to cook healthy food, how to store food, how to choose healthy food and what food should be avoid during pregnancy.
5. **Station #5 *the gym***: This station discusses the benefits of exercises during pregnancy, like swimming and yoga.
6. **Station 6 *the restaurant***: It provides instruction about checking restaurants hygiene and kind of food to be avoided by pregnant women such as uncooked seafood and sushi.
7. **Station #7 *the women’s beauty salon***: The content of this station focused on the effects of some cosmetics on the embryo, like hair dyes and hair treatments (protein and keratin chemicals).
8. **Station #8 *the mall***: It provides guidelines regarding clothes shopping for both the pregnant and her baby. It highlighted on how to choose good quality cloths and suitable sizes.
9. **Station #9 *the park***: This station promotes the importance of walking and practicing some healthy tips for example choosing comfortable shoes, drinking water, also avoiding smoking because of its potential dangers on pregnancy.
10. **Station #10 *the airport***: This is the last station and it highlights precautions to be considered during pregnancy when travelling, how to behave during the flight, and the most convenient time for traveling. It also emphasized on the importance of inform and her medical doctor about travelling plans.

It is worth mentioning that Saudi culture, dress, and language were considered during the design to make it more accepted by the patient.

#### Goal cards

**Fig 2:**
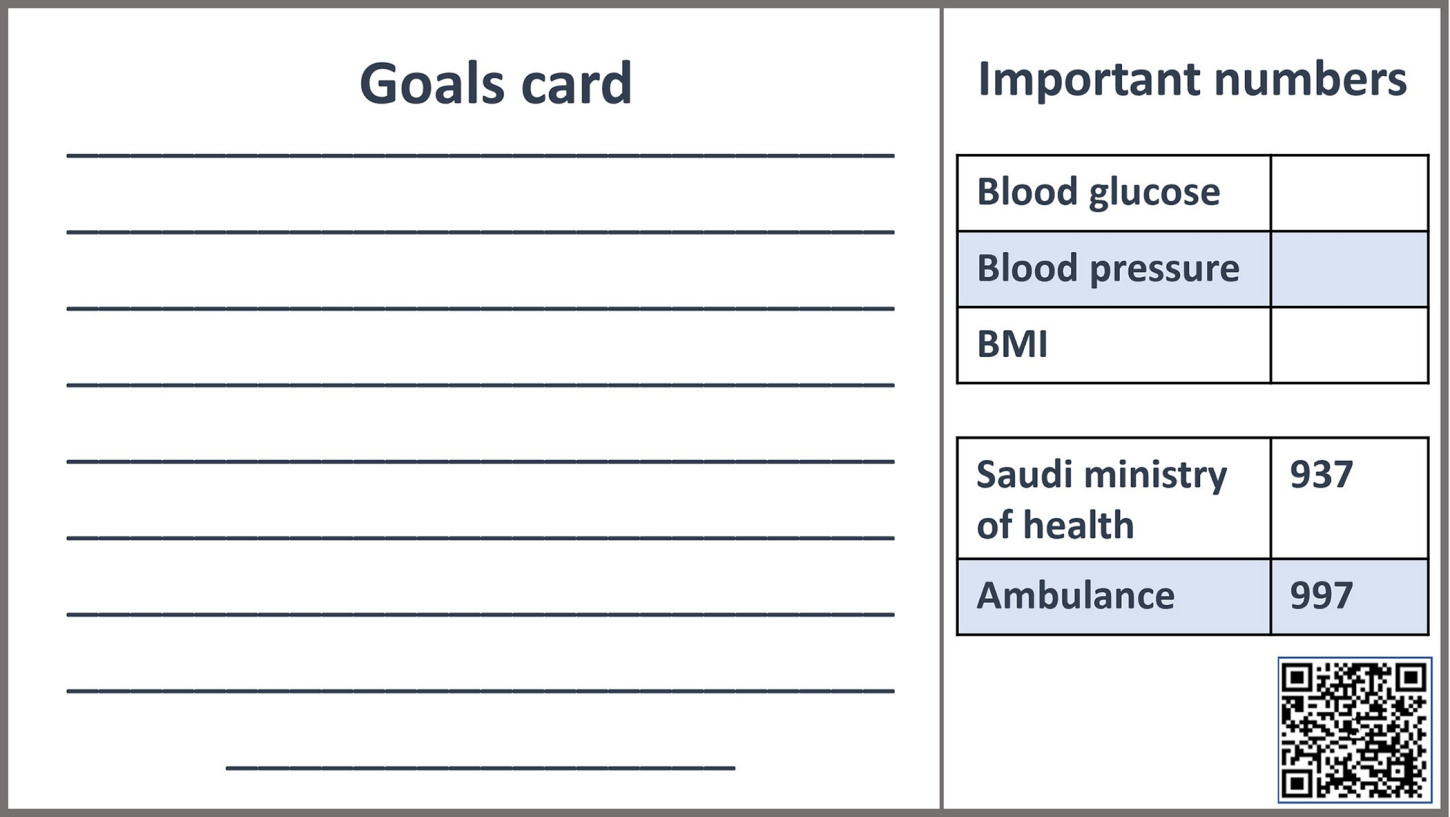
Goals card.

The goals card (Fig 2) was designed for patients to write down the goals they want to achieve. It also includes important phone numbers which might be needed such as Saudi Ministry of Health and Saudi Red Crescent. Furthermore, the goal cards contained important signs that pregnancy should maintain within normal ranges, such as blood pressure, blood glucose and body mass index. Additional information on pregnancy from Saudi Ministry of Health website was provided as a barcode.

#### Guide cards

**Fig 3:**
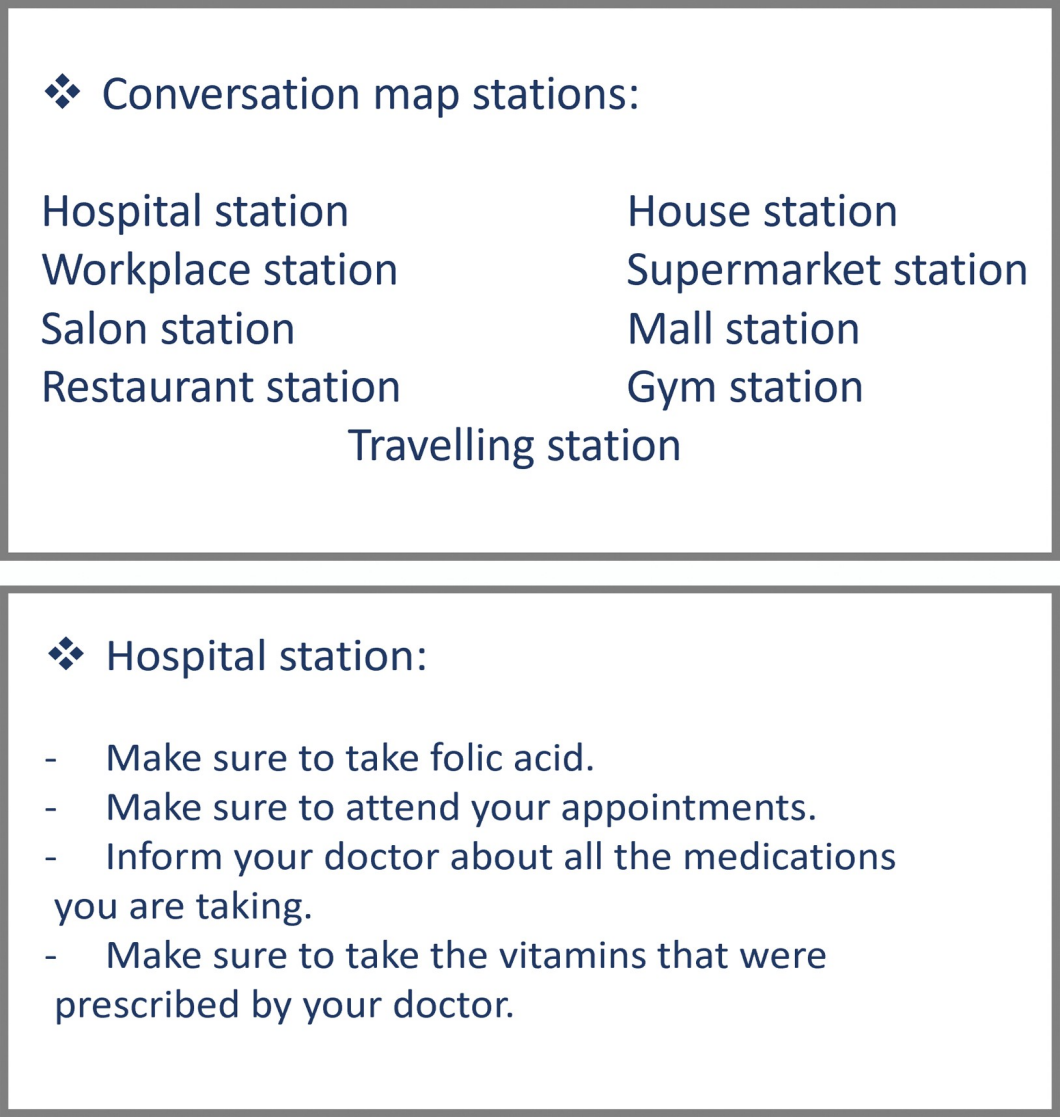
a Example 1 of guide card. b. Example 2 of guide card.

The guide cards were used as a supportive educational material (Fig 3A and 3B). They are given to the patients to obtain specific guidelines and contents of each station of the conversation map.

### Data collection

Conversation map training sessions were conducted by the researchers to educate health care providers. Alongside with it, goals and guide cards (Fig 2) were explained to them. Each session took about 15 minutes. The sessions were delivered for groups of a maximum of 5 health educators, based on their availability. During the session, the healthcare providers were given an explanation about the conversation map and its goals and stations.

After that, data was collected by self-administrated questionnaire. The questions were designed based on diffusion of innovation theory [11]. The questions were organized into four main sections as follows:

1. **Socio-demographic section** composed of questions on age, gender, specialty and the level of education.
2. **Health education services section** contained questions on numbers of years of experience, work settings, tools and techniques used when delivering health education sessions.
3. **Adoption of conversation map section** composed has three questions related to on having heard and/or used about the conversation map and how much time the participant thinks it takes to cover all the conversation map stations.
4. **Diffusion of innovation variables section:** A total of 23questions were directed toward relative advantage (6 questions), compatibility (5 questions), complexity (5 questions), trialability (3 questions) and observability (4 questions). The scoring of Likert scale was giving by numbers, strongly disagree = 1, disagree = 2, neutral = 3, agree = 4, strongly agree = 5. We used a specific formula for calculating the cutoff point, which was: maximum score minus minimum score, the difference was divided by 3, to have three categories: positive, neutral, and negative. At the end of the questionnaire, there was supported open question about additional comments to improve the conversation map.

### Reliability and validity

A pilot test was performed among 21 health care providers before the questionnaire was used for the study. We tested the face validity of the questionnaire through five experts in public health to modify and assure the quality of the questionnaire. Minor changes were recommended and applied to the questionnaire and the questionnaire was modified accordingly. The Cronbach’s alpha indicated that the questionnaire has a good reliability as it was equal to 0.8 that for the relative advantage, compatibility and observability were 0.8, and 0.7 for complexity and trialability.

### Statistical analysis

The JMP statistical software from SAS version 14 was used to perform data analysis [16].

Two independent sample t-test was used to compare the mean of diffusion of innovation variables by gender. One-way ANOVA test was used to compare the mean of diffusion of innovation variables section with the rest of socio-demographic section that were: age, specialty and level of education. The correlation test was used to assess the correlation between the diffusion of innovation variables with each other. A p-value was <0.05 was considered as significant.

## Results

**Table 1** summarizes the socio-demographic characteristics of the participants. Majority (77.3%) of them were female and more than two thirds (68.2%) were aged between 20 to less than 30 years. 30.7%, was allied health sciences specialties and 77.3% had bachelor’s degree.

**Table 1:** Sample characteristics (n = 88).

Characteristics	n	Percentage (%)
Gender Female Male	68 20	77.3 22.7
Age (years) 20 –<30 30 –< 40 years 40 –< 50 years ≥50	60 21 6 1	68.2 23.8 6.8 1.2
Specialty Allied health sciences Health education Medicine Nursing Social work Others	27 24 9 19 7 2	30.7 27.3 10.2 21.6 8.0 2.2
Level of education Diploma Bachelor Master	5 68 15	5.7 77.3 17.0
Years of experience in health education Internship Less than 3 years 3 –less than 6 years 6 –less than 9 years More than 9 years	27 14 19 10 18	30.7 15.9 21.6 11.4 20.5
In which setting do you apply your health education sessions? Inpatient Outpatient clinic Community and campaigns Health societies Others	50 66 44 28 1	56.8 75.0 50.0 31.8 1.1
Technique/s used while delivering health education* One to one counseling Group sessions Mass education Others	73 42 25 4	83.0 47.7 28.4 4.5
Tool/s used when delivering health education* Printable (pamphlets, brochures, posters… etc.) Digitals (video, power point… etc.) Conversation map Models Social media (Twitter, WhatsApp, Email… etc.) Others	64 53 11 28 38 11	72.7 60.2 12.5 31.8 43.2 12.2
Having ever heard about conversation map No Yes	73 15	83.0 17.0
Having ever used conversation map No Yes	77 11	87.5 12.5
The expected time to cover all stations if already used a conversation map 20 –less than 30 minutes 30 –less than 40 minutes Less than 20 minutes	3 4 2	33.3 44.4 22.3

*Participants were able to choose more than one answer.

In terms of health education services, 30.7%, of participants interns with. Three quarters (75%) were applying their health education sessions in outpatient clinics, and 83.0% used one to one counseling technique in applying health education. Printable tools such as pamphlets and brochures were most common as used by 72.7% of participants, while only 12.2% used conversation map and other tools. When asked about the adoption of conversation map, majority of the participants (83.0%) did not hear about it. Most of the participants were not using conversation map (87.50%). In addition, 44.4% of those who were using conversation map state that it would take 30 to less than 40 minutes is enough to cover all stations.

**Fig 4:**
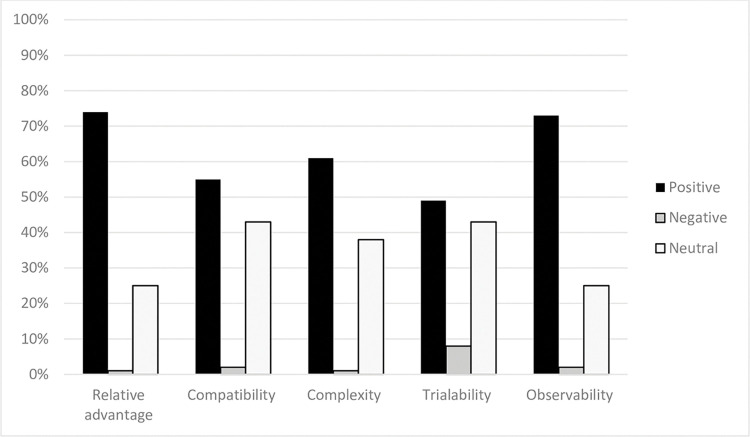
Distribution of participants (%) according to their total mean score of diffusion of innovation variables.

**Fig 4** represents that distribution of participants according to their scoring related to different variables (relative advantage, compatibility, complexity, trialability and observability) of the innovation diffusion theory on the use of the conversation map. In general, participants reported positive scoring with 74% for relative advantage, 55% for compatibility, 61% for complexity, 49% for trialability, and 73% for to observability.

**Table 2** shows the differences in diffusion of innovation variables and its relation to participants’ characteristics. No gender difference was found in relative advantage (p-value = 0.367). The mean of different variables was higher in all diffusion of innovation variables in females than males, but the difference is not statistically significant (p-value> 0.05). As for age groups, highest average was obtained for group age 40- <50 years for relative advantage and observability while highest average for compatibility, complexity, and trialability the highest average was obtained in participants aged from 50 years and more. No significant difference was obtained. Significant differences were obtained in both compatibility and trialability considering the health educators specialty, p = 0.03 and p = 0.027 respectively. The highest average was scored by social work specialist for compatibility and nursing for trialability. Diploma as a level of education showed the highest average in the studied variable of innovation theory compared to Bachelor and master’s degree (p-value >0.05).

**Table 2:** Differences in diffusion of innovation variables by participants’ characteristics.

	Relative advantage	Compatibility	Complexity	Trialability	Observability
**Gender** Female (n = 68) Male (n = 20) *P-value*	24.3±3.3 23.1±5.3 0.3674	18.5±3.2 17.6±4.4 0.408	19.3±2.7 19.0±4.1 0.787	11.2±2.0 10.5±3.4 0.358	15.3±2.4 14.9±3.3 0.577
**Age (years)** 20 –<30 (n = 60) 30 –< 40 (n = 21) 40 –< 50 (n = 6) ≥50 (n = 1) *P-value*	23.6±3.9 24.0±3.5 27.0±3.3 26.0 0.2092	17.9±3.4 19.0±3.8 19.6±3.7 20.0 0.42	19.2±3.0 19.3±3.0 18.3±3.6 20.0 0.9010	10.9±2.2 11.1±3.0 11.7±2.1 13.0 0.7764	15.0±2.6 15.2±2.7 17.0±2.0 0.16 0.3403
**Specialty** Medicine (n = 9) Nursing (n = 19) Health education (n = 24) Allied health sciences (n = 27) Social work (n = 7) Others (n = 2) *P-value*	23.1±7.1 24.8±3.2 24.5±3.5 23.1±3.5 23.9±2.3 25.0±1.4 0.6504	17.2±5.7 20.0±3.0 18.4±3.3 17.0±2.9 20.1±1.5 16.5±3.5 **0.0330**	18.6±5.6 19.8±3.3 19.7±2.8 18.3±2.0 20.4±1.9 18.5±0.7 0.3289	11.1±4.0 12.2±1.9 11.5±2.0 9.92±2.14 10.42±1.8 10.0±0.0 **0.0267**	14.8±4.8 16.1±2.8 15.1±2.3 14.7±2.0 15.4±0.3 14.5±0.7 0.6041
**Level of education** Diploma (n = 5) Bachelor (n = 68) Master (n = 15) *P-value*	25.8±2.7 23.9±4.0 23.7±3.4 0.5397	20.4±1.5 18.10±3.5 18.4±3.9 0.3674	20.2±2.9 19.2±3.0 18.8±3.1 0.6715	13.2±1.6 10.9±2.3 11.0±3.0 0.1096	17.4±1.9 15.1±2.6 14.9±2.5 0.1401

P-value was calculated by One-Way ANOVA test.

**Table 3** demonstrates the coefficient of the linear correlations (r) between diffusion of innovation variables was significantly positive (p-value <0.01). Strongest correlation was obtained between observability and relative advantage (r = 0.75) followed by observability and compatibility (0.73), and trialability (r = 0.73). This finding may provide some insight into the significance of being able to observe the benefits of the innovation, as well as the noticing the relative advantages, level of complexity and triability of the innovation. If a healthcare professional observed the benefits of the conversation map and believed that the map may be superior than any other educational tool they had used in the past, then they would improve the rate of adoption. Secondly, they will also value the conversation map if they have recognized the benefits of using it with their patients and feel it is simple to apply. Lastly, healthcare providers would value the conversation map more if they saw the benefits of putting it into practice after trying it on their own. This result could support the purpose of the study: when designing a conversation map for a healthcare provider, after the design process, education sessions for the healthcare provider should focus on observability while educating about other factors such as relative advantages, complexity, and triability in order to increase the conversation map’s adoption rate.

**Table 3:** Linear correlations (r) between the diffusion of innovation variables.

	Relative advantage	Compatibility	Complexity	Trialability	Observability
Relative advantage	1	--	--	--	--
Compatibility	0.68*	1	--	--	--
Complexity	0.59*	0.55*	1	--	--
Trialability	0.56*	0.64*	0.61*	1	
Observability	0.75*	0.73*	0.65*	0.73*	1

*Statistically high significant at p< 0.01.

## Discussion

We found that more than a quarter of the participants were interns and because the sample was a convenience sample method, the interns were readily available. Most of the participants were applying their health education sessions in outpatient clinics and given the opportunity to deliver effective and meaningful health education sessions. According to Skinner & Derryberry [17], health education has an effective opportunity to be applied in outpatient clinics.

The majority of the participants were female and most of the participants were under 30 years of age. Over a quarter of the participants were specialists in allied health sciences. The majority of the participants had a bachelor’s degree in their specialty. Additionally, the majority of the participants used one to one counseling techniques in applying health education sessions. This is aligned with the patient-centered techniques advocated by the Saudi Ministry of Health [18]. According to a previous study that assessed patients’ satisfaction in relation to health education sessions, it was found that one to one education technique is preferred when offering health education [18].

The majority of the participants were using printable tools such as pamphlets and brochures. In addition, printable and digital tools were equally used by participants in their health education sessions. This may be because printable and digital tools are easily accessible and available than other resources. The Saudi Ministry of Health website offers videos and brochures covering different health issues and these materials are available for printing and use [19].

We found that most of the participants have not used nor heard about the conversation mapping. This may be because it is relatively new in Saudi Arabia. According to a previous study, the diabetes conversation map was only used in about 110 countries regarding other issues [8]. A point which must be stressed, there is a lack of information about adopting the conversation map in Saudi Arabia for health education purposes, as by screening literature only one research study related to this topic was obtained in Saudi Arabia [20].

One of the objectives of this study was to measure the factors that influence the adoption of antenatal conversation mapping among health care providers in Riyadh, Saudi Arabia. The findings revealed that the total mean score of diffusion of innovation variables showed was in general high. A previous study conducted in Pakistan to assess the views and feedback about diabetes conversation mapping from trained health care providers concluded that the conversation map was a useful tool for educating diabetic patients [21].

The total mean score of diffusion of innovation variables in females was higher than males, the reason behind that was attributed to more female than male participants. The total mean score of relative advantage and observability was high in participants aged between 40 to less than 50 years, while the total mean score of compatibility, complexity, and trialability was high in participants aged from 50 years and more. On the other hand, the five diffusion of innovation variables were high in participants with a diploma degree, because the sample was small without a significant difference. According to a previous study conducted to assess the acceptance and resistance of new digital technologies in medicine, it was found that there are factors that can influence the acceptance of new technologies, such as hospital culture, location, and size of hospital [22].

Additionally, the total mean score of relative advantage was high among nursing specialist and health education, in addition to other specialties (not specified), although the findings are. And the total mean score of complexity variable was high among social work specialty, this may be because the number of participants was low in social work specialty. Also, the total mean score of observability variable was high in nursing specialty. According to a study conducted to assess the feedback about the conversation map from trained health care providers in Pakistan among different health specialties, they reported that their ability to educate patients with diabetes has increased [21]. In addition, the total mean score of compatibility and trialability variables was higher in nursing specialty than allied health sciences specialties, this may be because the conversation map was compatible with nurse education routine. Patient education is an important component of nursing competencies [23].

We found that diffusion of innovation variables had a positive moderate relationship with each other, and this may be attributed to the fact that diffusion of innovation variables influences the adoption of innovation. According to Rogers, the five diffusion of innovation variables had an effect on the adoption of innovation [11].

As a delimitation, we used antenatal care topic specifically for the care during pregnancy to present the conversation map. Limitations that we faced were the small sample size, due to the technique used to collect data which was the convenience sample method. We recognize that generalizability cannot be applied because the sample was not randomly selected, so it is not representative to the whole population. Also, access to the population data was limited to a predetermined time frame that could not be extended. For these reasons, researchers chose this study design. Other limitations were the conversation map training sessions that was conducted by the researchers to health care providers were presented each time by the four researchers, and this may have caused differences in understanding the conversation map. Also, the conversation map, goals cards. and guide cards were printed in the Arabic language, while some participants were English speakers. Furthermore, researchers did not ask who has used the conversation map regarding extent and experience in using it to assess the adoption rate.

## Conclusion

In conclusion, all of diffusion of innovation variables had a positive impression in the opinion of the participants. Most health education tools used by health educators were the printable and digital tools and most of the participants have not used nor heard about the conversation map. There was a significant difference in the overall mean score of compatibility and trialability characteristics between individuals from the nursing specialty and those from allied health sciences specializations. This may indicate that nurses feel the conversation map is congruent with their professional beliefs and norms, and that they can modify it to meet their patient education needs and context. Additionally, they feel that if they test it and experiment with it, the conversation map will help them embrace it more quickly in their patient education sessions. In addition, there was a highly significant difference between each variable of diffusion of innovation variables with each other. As per recommendations, the authors suggest the following:

1. Applying the conversation map on other health topics in Saudi Arabia and other Arabic-speaking countries is warranted.
2. Applying training sessions among health care providers about how to use and design the conversation map is necessary.
3. Measuring and evaluating the adoption rate of conversation mapping among health care providers on other health topics should be explored.
